# Supplementary material for: Determinants of sleep quality among pregnant women in a selected institution in the Southern province, Sri Lanka
Source: PLoS One. 2024 Jul 18;19(7):e0305388. doi: 10.1371/journal.pone.0305388 (PMC11257308; doi:10.1371/journal.pone.0305388)
Supplement: S1 Appendix — (PDF) [file pone.0305388.s001.pdf]

Determinants of sleep quality among pregnant women in Teaching hospital, Mahamodara: a cross sectional study

INSTRUCTIONS

Please fill this questionnaire on your own. Please consider about your current pregnancy when you are providing answers.

Part A: Socio-demographic data & current pregnancy

Date: ……………………   Serial No: …………………..

Following questions are about pregnant women’s socio-demographic data and current pregnancy details. Mark (×) for the best response in relevant questions. Write the suitable answers on the space given in other questions.

1. Age (Years)

   18 – 25 ☐

   26 – 33 ☐

   34 – 41 ☐

   ≥42 ☐

2. Marital status

   Married ☐

   Single ☐

   Widowed ☐

   Divorced/Separated ☐

3. Number of children up to now

- 0 ☐
- 1 – 3 ☐
- ≥ 4 ☐

4. Occupational status

- Employed ☐
- Unemployed ☐

5. Educational status

- Degree/higher degree ☐
- Diploma ☐
- Up to A/L ☐
- Up to O/L ☐
- Below than O/L ☐
- Never gone to a school ☐

6. Monthly income (Rs.)

- < 10 000 ☐
- 10 000 – 20 000 ☐
- 20 000 – 30 000 ☐
- 30 000 – 40 000 ☐
- ≥ 40 000 ☐

7. Body Mass Index (kg/m^2)

- Underweight (<18.5) ☐
- Normal weight (18.5 – 24.9) ☐
- Overweight (25 – 29.9) ☐
- Obese (≥30) ☐

8. Gestational age

- Week 1 – 12 ☐
- Week 13 – 28 ☐
- Week 29 – 40 ☐

Part B: Data about home and self-environment (Mark (×) for the best response/ responses

1. Your home

- Single, separated house ☐
- Rent house ☐
- Several families live in one house ☐
- Live in mother's or neighbor's house ☐
- Flat house ☐
- Other ☐

2. If your answer is "Other", please mention the type of house

....

3. Do you have any disturbing situations for sleep, around your home?

- Yes ☐
- No ☐

4. If your answer for above (3) question is “Yes”, what are those situations from below statements?

Loud noises inside the home ☐

Loud noises outside the home ☐

Shout noises of children/people ☐

Too much use of light bulbs inside the home ☐

Other ☐

……………………………………………………………….

5. For sleep at night,

Have a separate bed room ☐

All of family members sleep together in one place ☐

Other ☐

……………………………………………………………….

6. Before to sleep at night (10-15 minutes before to sleep), I,

Use mobile phones ☐

Watch television ☐

Do not use any of above mentioned things ☐

7. Do you use any comfortable equipment such as special mattresses, pillows or special bed preparations for more comfortable sleep, in the pregnancy period?

Yes ☐

No ☐

8. For a physical & mental comfort, prior to sleep I,

- Have a bath ☐
- Use comfortable, loose cloths ☐
- Listen to music ☐
- Do meditation ☐
- Do yoga exercises ☐
- Other ☐

……………………………………………

9. Do you believe that there is an action/situation or other disturbing event for your comfortable night sleep?

- Yes ☐
- No ☐

10. Do you have a good support from your husband, from your family members, friends, for your work as well as for house works?

- Have high level of support ☐
- Have normal level of support ☐
- Have very low level of support ☐
- No any support ☐
