# Supplementary material for: Determinants of sleep quality among pregnant women in a selected institution in the Southern province, Sri Lanka
Source: PLoS One. 2024 Jul 18;19(7):e0305388. doi: 10.1371/journal.pone.0305388 (PMC11257308; doi:10.1371/journal.pone.0305388)
Supplement: S2 Appendix — (PDF) [file pone.0305388.s002.pdf]

Pittsburgh Sleep Quality Index – English Format

INSTRUCTIONS:

The following questions relate to your usual sleep habits during the past month **only**. Your answersshould indicate the most accurate reply for the **majority** of days and nights in the past month.

Please answer all questions.

---

1. During the past month, what time have you usually gone to bed at night?

   BED TIME______________

2. During the past month, how long (in minutes) has it usually taken you to fall asleep each night?

   NUMBER OF MINUTES__________

3. During the past month, what time have you usually gotten up in the morning?

   GETTING UP TIME______________

4. During the past month, how many hours of **actual sleep** did you get at night? (This may bedifferent than the number of hours you spent in bed.)

   HOURS OF SLEEP PER NIGHT______________

For each of the remaining questions, check the one best response. Please answer all questions.

5. During the past month, how often have you had trouble sleeping because you…..

a) Cannot get to sleep within 30 minutes

Not during the Past month____	Less than once a week______	Once or twice a week ___	three or more times a week____

b) Wake up in the middle of the night or early morning

Not during the Past month____	Less than once a week___	Once or twice a week___	three or more times a week____

c) Have to get up to use the bathroom

Not during the Past month____	Less than once a week____	Once or twice a week______	three or more times a week____

d) Cannot breath comfortably

Not during the Past month____	Less than once a week____	Once or twice a week___	three or more times a week____

e) Cough or snore loudly

Not during the Past month____	Less than once a week_____	Once or twice a week______	three or more times a week____

f) Feel too cold

Not during the Past month____	Less than once a week_____	Once or twice a week______	three or more times a week____

g) Feel too hot

Not during the Past month____	Less than once a week_____	Once or twice a week____	three or more times a week____

h) Had bad dreams

Not during the Past month____ | Less than once a week_____ | Once or twice a week____ | three or more times a week____

i) Have pain
j)

Not during the Past month____ | Less than once a week_____ | Once or twice a week_______ | three or more times a week____

k) Other reason(s), please describe______________________________________

How often during the past month have you had trouble sleeping because of this?

Not during the Past month____ | Less than once a week___ | Once or twice a week______ | three or more times a week____

6. During the past month, how would you rate your sleep quality overall?

Very good_________

Fairly good________

Fairly bad_________

Very bad__________

7. During the past month, how often have you taken medicine to help you sleep (prescribed or"over the counter")?

Not during the Past month____ | Less than once a week_____ | Once or twice a week_______ | three or more times a week_____

8. During the past month, how often have you had trouble staying awake while driving, eatingmeals, or engaging in social activity?

Not during the Past month____	Less than once a week_____	Once or twice a week___	three or more times a week____

9. During the past month, how much of a problem has it been for you to keep up enoughenthusiasm to get things done?

No problem at all_______________

Only a very slight problem_____________

Somewhat of a problem_______________

A very big problem_________________

10. Do you have a bed partner or roommate?

No bed partner or room mate___________

Partner/roommate in other room____________

Partner in same room, but not same bed__________

Partner in same bed____________________

If you have a roommate or bed partner, ask him/her how often in the past month youhave had

a) Loud snoring

Not during the Past month____	Less than once a week___	Once or twice a week______	three or more times a week____

b) Long pauses between breaths while asleep

Not during the Past month____	Less than once a week___	Once or twice a week____	three or more times a week____

c) Legs twitching or jerking while you sleep

Not during the Past month____	Less than once a week___	Once or twice a week______	three or more times a week____

d) Episodes of disorientation or confusion during sleep

Not during the Past month____	Less than once a week___	Once or twice a week______	three or more times a week____

e) Other restlessness while you sleep; please describe ________________________-
____

Not during the Past month____	Less than once a week____	Once or twice a week____	three or more times a week____

<u>Edinburgh Postnatal Depression Scale (EPDS) – English Format</u>

As you are pregnant or have recently had a baby, we would like to know how you are feeling. Please checkthe answer that comes closest to how you have felt <u>IN THE PAST 7 DAYS,</u> not just how you feel today.

Here is an example, already completed.I have felt happy:

- Yes, all the time
- Yes, most of the time
- No, not very often
- No, not at all

In the past 7 days:

1) I have been able to laugh and see the funny side of things
    - As much as I always could
    - Not quite so much
    - Definitely not so much now
    - Not at all
2) I have looked forward with enjoyment to things
    - As much as I ever did
    - Rather less than I used to
    - Definitely less than I used to
    - Hardly at all
3) I have blamed myself unnecessarily when things went wrong
    - Yes, most of the time
    - Yes, some of the time
    - Not very often
    - No, never
4) I have been anxious or worried for no good reason
    - No, not at all
    - Hardly ever
    - Yes, sometimes
    - Yes, very often

5) I have felt scared or panicky for no very good reason
    - Yes, quite a lot
    - Yes, sometimes
    - No, not much
    - No, not at all
6) Things have been getting on top of me
    - Yes, most of the time I haven't been able to cope at all
    - Yes, sometimes I haven't been coping as well as usual
    - No, most of the time I have coped quite well
    - No, I have been coping as well as ever

7) I have been so unhappy that I have had difficulty sleeping

- Yes, most of the time
- Yes, sometimes
- Not very often
- No, not at all

8) I have felt sad or miserable

- Yes, most of the time
- Yes, quite often
- Not very often
- No, not at all

9) I have been so unhappy that I have been crying

- Yes, most of the time
- Yes, quite often
- Only occasionally
- No, never

10) The thought of harming myself has occurred to me

- Yes, quite often
- Sometimes
- Hardly ever
- Never
