# Supplementary material for: Determinants of sleep quality among pregnant women in a selected institution in the Southern province, Sri Lanka
Source: PLoS One. 2024 Jul 18;19(7):e0305388. doi: 10.1371/journal.pone.0305388 (PMC11257308; doi:10.1371/journal.pone.0305388)
Supplement: S3 Appendix — (PDF) [file pone.0305388.s003.pdf]

## <u>Generalized Anxiety Disorders-7 scale – English Format</u>

Mark (×) on the relevant answer, in the relevant column.

| Over the last 2 weeks, how often have you been bothered by the following problems? | Not at all sure | Several days | Over half the days | Nearly every day |
|---|---|---|---|---|
| 1. Feeling nervous, anxious, or on edge | 0 | 1 | 2 | 3 |
| 2. Not being able to stop or control worrying | 0 | 1 | 2 | 3 |
| 3. Worrying too much about different things | 0 | 1 | 2 | 3 |
| 4. Trouble relaxing | 0 | 1 | 2 | 3 |
| 5. Being so restless that it's hard to sit still | 0 | 1 | 2 | 3 |
| 6. Becoming easily annoyed or irritable | 0 | 1 | 2 | 3 |
| 7. Feeling afraid as if something awful might happen | 0 | 1 | 2 | 3 |

Thank You for Your Time and Answers!
