# Supplementary material for: Determinants of sleep quality among pregnant women in a selected institution in the Southern province, Sri Lanka
Source: PLoS One. 2024 Jul 18;19(7):e0305388. doi: 10.1371/journal.pone.0305388 (PMC11257308; doi:10.1371/journal.pone.0305388)
Supplement: S4 Appendix — (PDF) [file pone.0305388.s004.pdf]

Information sheet

Determinants of sleep quality among pregnant women in Teaching Hospital, Mahamodara: A cross-sectional study

I am Ms. M. S. K. Peiris, an undergraduate student, Department of Nursing, Faculty of Allied Health Sciences, University of Ruhuna. As a component of my BSc. Degree in nursing, I have to conduct a research project. I will conduct this research with under the supervision of Dr. (Mrs.) S. M. T. D. Sundarapperuma, Senior Lecturer, Department of Nursing, Faculty of Allied Health Sciences, University of Ruhuna. This research proposal has been reviewed and approved by the Ethics Review Committee of the Faculty of Allied Health Sciences, University of Ruhuna. Permission for this research study has been obtained from the Director of the Teaching Hospital, Mahamodara and Consultants, Specialist Doctors, Special grade nursing officers of relevant antenatal wards and antenatal clinics.

1. Purpose of the study

To assess determinants of sleep quality among pregnant women in Teaching Hospital, Mahamodara, determine the prevalence of poor sleep quality, identify the difference in sleep quality in each trimester and identify the psychological disturbances encountered with pregnant mothers due to poor sleep quality.

2. Procedure

Participants to this study will be invited to pregnant women in antenatal wards and antenatal clinics in teaching hospital, Mahamodara. A self-administered questionnaire will be used to collect your demographic data, current pregnancy and home and self-environment data. PSQI use to collect data about sleep quality and disturbances over one month interval and EPDS and GAD-7 item scale use to collect data about prenatal psychological disturbances. I will give clear instructions to you about filling the questionnaire, PSQI and EPDS and GAD-7 items scales. Then I will collect completed questionnaire, PSQI and EPDS and GAD-7 items scales.

3. Participant selection

All the pregnant women in antenatal wards and antenatal clinics, who are aged more than 18 years old, who are willing to participate in the teaching hospital, Mahamodara and who can write, read, and understand of Sinhala or English language will be included to study after obtaining their volunteer consent form. If the pregnant women, who are not willing to participate and who have severe psychiatric illnesses will be excluded from the study.

4. Benefits and results

There may not be any benefits or risk for you. However your participation may help us to find answers to the research objectives and the results of this study can be used to assess the determinants of sleep quality among pregnant women. There may not be any benefit to the society at this stage of the research, but those results may fruitful to future generations.

5. Reimbursement

You will not be given any other money or gifts to take part of this study.

6. Confidentiality

All information obtained during the study is strictly confidential. Your anonymity will be protected all the time. You will be identified by a serial number. The collected data regarding you will handle by researchers only. Any information about you will have a number with it instead of your name. Only the researchers will know your serial number. We will publish the results in order that other interested people may learn from our results and confidential information will not be shared.

7. Termination of the study participation

Before you participate for the study, you can talk to anyone you feel comfortable with about the study. You do not have to take part of the study if you do not wish to participate. You will be free to withdraw at any time during the study without giving any reason. It is your choice and all of your rights will be highly respected.

8. Clarification

If you have any question to clarify, you may ask from me now or later. If you need more information you can ask from me at any time. All contact details of me, chief supervisor and Ethics Review Committee will be mentioned here.

Name of the investigator – M. S. K. Peiris

Email Address – kaushinipeiris@gmail.com

Contact number – 071 7637193

Name of the chief supervisor – Dr. (Mrs.) S. M. T. D. Sundarapperuma, Senior Lecturer, Department of Nursing, Faculty of Allied Health Sciences, University of Ruhuna.

Email Address – chamudi2006@yahoo.com

Contact number – 071 3186524

Contact details of Ethics Review Committee – Dr. H. H. Peiris, Chairperson, Ethics Review Committee, Faculty of Allied Health Sciences, University of Ruhuna.

Email Address – ethics.fahs@gmail.com

Contact number – 091 2234676
