# Supplementary material for: Determinants of sleep quality among pregnant women in a selected institution in the Southern province, Sri Lanka
Source: PLoS One. 2024 Jul 18;19(7):e0305388. doi: 10.1371/journal.pone.0305388 (PMC11257308; doi:10.1371/journal.pone.0305388)
Supplement: S5 Appendix — (PDF) [file pone.0305388.s005.pdf]

<u>Volunteer consent form</u>

This informed consent form is for pregnant women who have admitted to the …………….-..ward/clinic in Teaching hospital, Mahamodara.

I am …………………... I am inviting you to participate in the study on “Determinants of sleep quality among pregnant women in Teaching hospital, Mahamodara: a cross sectional study”. The information sheet has already provided about the study.

<u>Statement of the participant</u>

I………………………………………….. (Name of the participant) have read the information sheet and understand.

The study has been explained to me and I understood,

- What the research study involves
- Then I can withdraw any time and it will not affect me adversely in any manner.

Therefor I agree to give all the necessary information related to this study.

Signature of the participant………………………..
