# Supplementary material for: Determinants of sleep quality among pregnant women in a selected institution in the Southern province, Sri Lanka
Source: PLoS One. 2024 Jul 18;19(7):e0305388. doi: 10.1371/journal.pone.0305388 (PMC11257308; doi:10.1371/journal.pone.0305388)
Supplement: Attachment — Submitted filename: PONE-D-24-00504_reviewer.pdf [file pone.0305388.s006.pdf]

PLOS ONE

Determinants of sleep quality among pregnant women in teaching hospital mahamodara, Galle, Sri Lanka

--Manuscript Draft--

**Manuscript Number:**	PONE-D-24-00504
**Article Type:**	Research Article
**Full Title:**	Determinants of sleep quality among pregnant women in teaching hospital mahamodara, Galle, Sri Lanka
**Short Title:**	Determinants of sleep quality among pregnant women
**Corresponding Author:**	THAMUDI Darshi Sundarapperuma<br>University of Ruhuna<br>GALLE, SRI LANKA
**Keywords:**	Sleep quality, pregnant mothers, Depression, PSQI
**Abstract:**	Sleep is a natural physiological process that is essential for the physical and mental well-being of pregnant women and their fetuses. Sleep disturbances cause significant morbidity and mortality in pregnant women and burden fetal growth. The purpose of the present study was to assess determinants of sleep quality among pregnant women in teaching hospitals, Mahamodara. This hospital-based descriptive cross-sectional study was carried out with 245 antenatal mothers, selected using a simple random sampling method. A pretested self-administered questionnaire was used to collect data. Data was analyzed using one-way ANOVA and independent samples t-test and p<0.05 was considered as statistically significant. Participants of 60.8 had good sleep quality and did not experience depression and anxiety. Gestational age and increasing maternal age were significantly associated with sleep quality. Age between 34 – 41 years and third-trimester women had higher rates of poor sleep quality. Varying quality of sleep was identified among three trimesters with subjective sleep quality, sleep latency, habitual sleep efficiency, and sleep disturbances. In comparison with the first and second trimester, pregnant women in the third trimester had higher score of global PSQI (5.22 ± 2.35), subjective sleep quality (1.23 ± 0.70), sleep latency (1.25 ± 0.86), habitual sleep efficiency (0.14 ± 0.43), and sleep disturbances (1.39 ± 0.58). There was a significant association between antenatal depression, anxiety with sleep quality. As a recommendation, it is better to develop a validated tool to assess sleep quality, especially for Sri Lanka, and plan awareness programs for quality sleeping during the pregnancy period.
**Order of Authors:**	THAMUDI Darshi Sundarapperuma
	Kaushini Peiris, BSc.Nursing
**Additional Information:**	
**Question**	**Response**
**Financial Disclosure**<br><br>Enter a financial disclosure statement that describes the sources of funding for the work included in this submission. Review the submission guidelines for detailed requirements. View published research articles from *PLOS ONE* for specific examples.<br><br>This statement is required for submission and **will appear in the published article** if the submission is accepted. Please make	The author(s) received no specific funding for this work.

Powered by Editorial Manager® and ProduXion Manager® from Aries Systems Corporation

sure it is accurate.<br><br>**Funded studies**<br>Enter a statement with the following details:<br>• Initials of the authors who received each award<br>• Grant numbers awarded to each author<br>• The full name of each funder<br>• URL of each funder website<br>• Did the sponsors or funders play any role in the study design, data collection and analysis, decision to publish, or preparation of the manuscript?<br><br>Did you receive funding for this work?	
**Competing Interests**<br><br>Use the instructions below to enter a competing interest statement for this submission. On behalf of all authors, disclose any competing interests that could be perceived to bias this work—acknowledging all financial support and any other relevant financial or non-financial competing interests.<br><br>This statement is required for submission and **will appear in the published article** if the submission is accepted. Please make sure it is accurate and that any funding sources listed in your Funding Information later in the submission form are also declared in your Financial Disclosure statement.<br><br>View published research articles from *PLOS ONE* for specific examples.	The authors have declared that no competing interests exist.

**NO authors have competing interests**<br><br>Enter: *The authors have declared that no competing interests exist.*<br><br>**Authors with competing interests**<br><br>Enter competing interest details beginning with this statement:<br><br>*I have read the journal's policy and the authors of this manuscript have the following competing interests: [insert competing interests here]*<br><br>* typeset	
**Ethics Statement**<br><br>Enter an ethics statement for this submission. This statement is required if the study involved:<br><br>• Human participants<br>• Human specimens or tissue<br>• Vertebrate animals or cephalopods<br>• Vertebrate embryos or tissues<br>• Field research<br><br>Write "N/A" if the submission does not require an ethics statement.<br><br>General guidance is provided below. Consult the submission guidelines for detailed instructions. **Make sure that all information entered here is included in the Methods section of the manuscript.**	Ethical clearance was obtained from the Ethical Review Committee, Faculty of Allied Health Sciences, University of Ruhuna(No:59.11.2021).<br>Informed written consent was taken from all participants.

Powered by Editorial Manager® and ProduXion Manager® from Aries Systems Corporation

**Format for specific study types**<br><br>**Human Subject Research (involving human participants and/or tissue)**<br>• Give the name of the institutional review board or ethics committee that approved the study<br>• Include the approval number and/or a statement indicating approval of this research<br>• Indicate the form of consent obtained (written/oral) or the reason that consent was not obtained (e.g. the data were analyzed anonymously)<br><br>**Animal Research (involving vertebrate animals, embryos or tissues)**<br>• Provide the name of the Institutional Animal Care and Use Committee (IACUC) or other relevant ethics board that reviewed the study protocol, and indicate whether they approved this research or granted a formal waiver of ethical approval<br>• Include an approval number if one was obtained<br>• If the study involved *non-human primates*, add *additional details* about animal welfare and steps taken to ameliorate suffering<br>• If anesthesia, euthanasia, or any kind of animal sacrifice is part of the study, include briefly which substances and/or methods were applied<br><br>**Field Research**<br><br>Include the following details if this study involves the collection of plant, animal, or other materials from a natural setting:<br>• Field permit number<br>• Name of the institution or relevant body that granted permission	
**Data Availability**<br><br>Authors are required to make all data underlying the findings described fully available, without restriction, and from the time of publication. PLOS allows rare exceptions to address legal and ethical concerns. See the PLOS Data Policy and FAQ for detailed information.	Yes - all data are fully available without restriction

Powered by Editorial Manager® and ProduXion Manager® from Aries Systems Corporation

A Data Availability Statement describing where the data can be found is required at submission. Your answers to this question constitute the Data Availability Statement and **will be published in the article**, if accepted.<br><br>**Important:** Stating 'data available on request from the author' is not sufficient. If your data are only available upon request, select 'No' for the first question and explain your exceptional situation in the text box.<br><br>Do the authors confirm that all data underlying the findings described in their manuscript are fully available without restriction?	
**Describe where the data may be found in full sentences. If you are copying our sample text, replace any instances of XXX with the appropriate details.**<br><br>• If the data are **held or will be held in a public repository**, include URLs, accession numbers or DOIs. If this information will only be available after acceptance, indicate this by ticking the box below. For example: *All XXX files are available from the XXX database (accession number(s) XXX, XXX.).*<br>• If the data are all contained **within the manuscript and/or Supporting Information files**, enter the following: *All relevant data are within the manuscript and its Supporting Information files.*<br>• If neither of these applies but you are able to provide **details of access elsewhere**, with or without limitations, please do so. For example:<br><br>*Data cannot be shared publicly because of [XXX]. Data are available from the XXX Institutional Data Access / Ethics Committee (contact via XXX) for researchers who meet the criteria for access to confidential data.*<br><br>*The data underlying the results presented in the study are available from (include the name of the third party*	The data used to support the findings of this study are available from the corresponding author upon request.

and contact information or URL).<br>• This text is appropriate if the data are owned by a third party and authors do not have permission to share the data.<br><br>* typeset	
Additional data availability information:	

Manuscript

Click here to access/download;Manuscript;Revised PloS one article 1 - new.docx

Determinants of sleep quality among pregnant women in teaching hospital mahamodara, Galle, Sri Lanka

Determinants of sleep quality among pregnant women

Peiris M.S.K[1] , Sundarapperuma T.D[1*]

[1] Department of Nursing, Faculty of Allied Health Sciences, University of Ruhuna, Sri Lanka

[*]Corresponding author

E-mail: Thamudids@ahs.ruh.ac.lk (TD)

Both authors substantially contributed to all of the followings: (1) the conception and design of the study (2) analysis and interpretation of data, (3) drafting the article and revising it and, (4) final approval of the version to be submitted. In addition, author 1 mainly collected data

Abstract

Sleep is a natural physiological process that essential for the physical and mental wellbeing of pregnant women and their fetuses. Sleep disturbances cause significant morbidity and mortality in pregnant women and burden to fetal growth. The purpose of the present study was to assess determinants of sleep quality among pregnant women in teaching Hospital, Mahamodara. This hospital-based descriptive cross-sectional study was carried out with 245 antenatal mothers, selected using simple random sampling method. Pretested self-administered questionnaire was used to collect data. Data was analyzed using one-way ANOVA and independent samples t-test and $p<0.05$ was considered as statistically significant. Participants of 60.8 had good sleep quality and did not experience depression and anxiety. Gestational age and increasing maternal age were significantly associated with sleep quality. Age between 34 – 41 years and third trimester women had higher rates of poor sleep quality. Varying quality of sleep was identified among three-trimesters with subjective sleep quality, sleep latency, habitual sleep efficiency and sleep disturbances. Comparison with the first and second trimester, pregnant women in third trimester had higher score of global PSQI (5.22 ± 2.35), subjective sleep quality (1.23 ± 0.70), sleep latency (1.25 ± 0.86), habitual sleep efficiency (0.14 ± 0.43), and sleep disturbances (1.39 ± 0.58). There was a significant association between antenatal depressions, anxiety with sleep quality. As a recommendation, it is better to develop a validated tool to assess sleep quality especially for Sri Lanka and planning awareness programs of quality sleeping during pregnancy period.

Introduction

Sleep is one of the most important basic needs of the human being which helps to maintain metabolic activities effectively [1]. Sleep quality is a measurement of indicating how well people are sleeping. Basically, there are four elements use to measure sleep quality such as sleep latency, sleep waking, wakefulness and sleep efficiency [2]. Pregnancy as a unique period of a woman's life hence quality sleep is an essential requirement for a healthy pregnancy to conserve energy for delivery process with 7 – 9 hour sleep [3, 4].

Typically, pregnancy creates significant physiologically, anatomically and biochemically changes, and these changes may affect physically and emotionally to the

women's life which leads to sleep disturbances [5]. Enlarging abdomen, heartburn, frequent movements of the fetus are more commonly affect to each trimesters can be the reasons for a frequent awakening from the sleep and poor sleep quality of a pregnant woman [6]. Also the relationship between sleep quality and psychological disturbances can cause to maternal morbidity and neonatal birth complications [7].

There is dearth of studies have been conducted about sleep quality among pregnant women in Asian countries including Sri Lanka. A China study reported that 87% mothers experienced sleep disorders during pregnancy period while prenatal depression, age and gestational age had found as contributing factors for the sleep quality [5]. The studies done in other countries reveal higher prevalence of poor sleep quality and similar results has found as above study [8, 9]. Further, none of the reported studies in the south Asian region had compared the difference in sleep quality among 3 trimesters and neither of them had identified associated factors for the poor quality of sleep during each trimester. Therefore, objectives of this study were to determine prevalence of poor sleep quality, identify factors associated with poor sleep quality, compare the difference in sleep quality among three trimesters and identify the psychological disturbances encountered with pregnant mothers due to poor sleep quality in Teaching Hospital Mahamodara (THM), Galle.

Materials and Methods

Study setting and study population

A Hospital-based descriptive cross-sectional study was carried out in antenatal clinics and antenatal wards at Teaching Hospital Mahamodara. The study population consisted of all pregnant mothers who were registered in THM.

Eligibility criteria

Pregnant women who could understand Sinhala or English and who were willing to participate were included to the study after provide detailed information and take the consent. Pregnant

women who were not willing to participate and who had severe psychiatric illnesses were excluded from the study.

Sample size determination and sampling procedure

The sample size was calculated by using the formula suggested by Lowanga & Lameshow in 1991 with 1.96 as standard normal deviation for the chose confidence level, 83% as expected proportion of subject with characteristics, based on the research study of public health center in Yogyakarta, Indonesia [1] and another research study of determinants of sleep quality among pregnant women in China [5]. Accordingly, the required sample size was 217. A further adjustment to the sample size was made considering non-response or incomplete response rate of 10%, making the final sample size 242. Simple random sampling method used to select mothers from antenatal wards and antenatal clinics by taking a list of all mothers from the register of the particular antenatal ward or clinic and selected the individual who belonged to the even number.

Data collection and management

A self-administered questionnaire which contained four parts were used to collect data. These parts were pre-tested self-developed questionnaire to assess socio-demographic details. Validated Sinhala versions of Pittsburg Sleep Quality Index (PSQI) to collect data related to sleep quality, Edinburgh Postnatal Depression Scale (EPDS) to screen depression in antenatal women and Generalized Anxiety Disorder 7 scale (GAD-7) to screen anxiety level. The average duration of the questionnaire was 10 minutes. Informed written consent was taken after explaining the objective of the study. Ethical clearance was obtained from the Ethical Review Committee, Faculty of Allied Health Sciences, University of Ruhuna.

Operational definitions

'Sleep quality' was defines as an individual's self-satisfaction with all aspects of the sleep experience. 'Maternal anxiety' was defined as a feeling worry, nervousness or unease about something with an uncertain outcome during pregnancy period. 'Maternal depression' was

defined as a mood disorder that causes a persistent feeling of sadness and loss of interest during pregnancy period.

Data analysis

Data was analyzed using Statistical Package for Social Sciences (SPSS) 25 version software. Descriptive statistics such as mean (SD), median (IQR) and frequencies (%) used to describe level of psychosocial disturbances of study the participants. Normally distributed data was further analyzed for inferences, using ANOVA and independent samples t-test. Chi-square and correlation analysis were used to identify the associations between independent and dependent variables. P value less than 0.05 ($p<0.05$) was considered as statistically significant.

Ethics approval and consent to participate

Ethical clearance was obtained from the Ethical Review Committee, Faculty of Allied Health Sciences, University of Ruhuna (No: 59.11.2021). Informed written consent was taken from all participants after providing detailed information of purpose of the research study, advantages, results, confidentiality, and methods of the research. Confidentiality of all information was kept properly. The data were collected from 12th July to 21st July, 2022.

Results

Mean age of the pregnant women were 30 years. Majority of subjects were married, unemployed and education up to O/L with monthly income between 30,000 – 40,000 rupees. Most of the pregnant women were in the third trimester and primi mothers. Among the sample, most of mothers had children between 1 - 3 children. Majority of pregnant women had single/separated type of house. Concerning the type of bed room, most of them had a separate bed room for sleep at night while the majority of mothers had high level of family support for their day to day works. Their mean BMI was 18.5 to 24.9 (Table 1).

Table 1. Association of basic factors with the sleep quality among antenatal mothers

Variables	Frequencies (%)	Subcategories	Mean & SD	df	F-test	p-value

Age in Years (**)	62 (25.3)	18 – 25	4.73 ± 2.44	(3,241)	3.96	**0.01**
	126 (51.4)	26 – 33	4.52 ± 2.07			
	55 (22.4)	34 – 41	5.76 ± 2.50			
	2 (0.8)	≥42	5.00 ± 0.00			
Occupational Status (*)	66 (26.9)	Employed	4.92 ± 2.04	243	2.68	0.10
	179 (73.1)	Unemployed	4.82 ± 2.40			
Marital status (*)	242 (98.8)	Married	4.88 ± 2.30	243	1.01	0.31
	3 (1.2)	Unmarried	2.66 ± 1.52			
Educational Status (**)	18 (7.3)	Degree/higher degree	4.27 ± 1.64	(1,243)	0.08	0.51
	16 (6.5)	Diploma	4.94 ± 2.08			
	12 (4.9)	Other vocational programs	5.08 ± 2.71			
	77(31.4)	Up to A/L	5.01 ± 2.54			
	106 (43.3)	Up to O/L	4.93 ± 2.15			
	16 (6.5)	Below than O/L	3.94 ± 2.67			
BMI (kg/m^2) (**)	33 (13.5)	Underweight (<18.5)	5.09 ± 2.32	(3,241)	1.63	0.18
	178 (72.7)	Normal weight (18.5-24.9)	4.71 ± 2.29			
	31 (12.7)	Overweight (25-29.9)	5.12 ± 2.12			
	3 (1.2)	Obese (≥30)	7.33 ±4.04			
Gestational Age (**)	55 (22.4)	Week 1 – 12	4.00 ± 1.89	(2,242)	5.47	**0.01**
	72 (29.4)	Wee 13 – 26	4.90 ± 2.36			
	118 (48.2)	Week 27 - 40	5.22 ± 2.35			
Number of children (**)	99 (40.4)	0	4.81 ± 2.41	(2,242)	0.03	0.96
	140 (57.1)	1 – 3	4.88 ± 2.23			
	6 (2.4)	≥4	4.83 ± 2.64			

(*) used Independent sample T-test

(**) used one-way ANOVA test

This study showed that there were 39.2% had poor sleep quality while 60.8% had good sleep quality with mean global PSQI score 4.8531 (SD = 2.3053) and majority of participants had fairly good sleep quality during the last month of pregnancy period according to the PSQI results. There were majority of pregnant women had taken less than 15 minutes to

fall asleep each night during the past month while most of pregnant women had more than 7 hours of sleep during the last month.

Frequency distribution of sleep disturbances according to the PSQI showed that majority of participants had disturbed sleep three or more times a week due to wake up in the middle of the night and get up to use the bathroom during the last month as well as most of pregnant women had not any prescribed or over the counter medicines to help them in sleep. Daytime dysfunction over the past month according to the PSQI indicated most of participants had not trouble with staying awake while driving, eating meals, or engaging in social activity during the last month while 69% of mothers had not any problem to keep up enthusiasm to get things done (Table 2).

Table 2. Frequency distribution of associated factors according to the PSQI

Variable	Subcategories	Frequencies (%)
**How long (in minutes) has it taken you to fall asleep each night?**	<15 minutes (0)	160 (65.3)
	16 – 30 minutes (1)	66 (26.9)
	31 – 60 minutes (2)	15 (6.1)
	>60 minutes (3)	04 (1.6)
**During the past month, how often have you had trouble sleeping because you cannot get to sleep within 30 minutes?**	Not during the past month	97 (39.6)
	Less than once a week	54 (22.0)
	Once or twice a week	27 (11.0)
	Three or more times a week	67 (27.3)
**How many hours of actual sleep did you get at night?**	>7 hours (0)	189 (77.1)
	6 – 7 hours (1)	37 (15.1)
	5 – 6 hours (2)	18 (7.3)
	<5 hours (3)	01 (0.4)
**Habitual sleep efficiency=** $\frac{\textit{Total of hours asleep}}{\textit{Total of hours in bed}} \times 100$	>85% (0)	213 (86.9)
	75% – 84% (1)	27 (11.0)
	65% – 74% (2)	04 (1.6)
	<65% (3)	01 (0.4)

**During the past month, how often have you taken medicine (prescribed or 'over the counter') to help you sleep?**	Not during the past month (0)	189 (77.1)
	Less than once a week (1)	37 (15.1)
	Once or twice a week (2)	18 (7.3)
	Three or more times a week (3)	01 (0.4)

Association of basic factors with the sleep quality among antenatal mothers during the last month showed that there was statistically significant difference between global PSQI score and maternal age. Post hoc analysis results indicated that there was significant association between 26 – 33 years group with 34 – 41 years group. Overall global PSQI score indicated significant difference with gestational age and there was a significant association between 1st trimester with 3rd trimester. Also there was no statistically significant difference between the global PSQI score with number of children, educational status, marital status, occupational status and BMI category (Table 1).

Difference of sleep quality among three trimesters showed that pregnant women in third trimester had higher score of global PSQI, subjective sleep quality, sleep latency, habitual sleep efficiency, and sleep disturbances than first and second trimester. When compared with third trimester, pregnant women in second trimester had higher score of sleep duration, habitual sleep efficiency and daytime dysfunction. There was no statistically significant differences across trimesters in the following PSQI component scores: sleep duration, use of sleeping medication, and daytime dysfunction (Table 3). Association of psychological disturbances and sleep quality shows that there was a significant association between antenatal depressions and anxiety signs with sleep quality during antenatal period (Table 4).

Table 3. PSQI score and the differences among three trimesters

**Components**	**Gestational age**	***F***	***P***

	Mean score (n=245)	First trimester (n=55)	Second trimester (n=72)	Third trimester (n=118)		
**Global PSQI score**	4.85 ± 2.30	4.00 ± 1.89	4.90 ± 2.36	5.22 ± 2.35	5.47	0.005
**Component score**						
Subjective sleep quality	1.11 ± 0.66	0.91 ± 0.55	1.08 ± 0.64	1.23 ± 0.70	4.53	0.012
Sleep latency	1.06 ± 0.89	0.78 ± 0.81	0.98 ± 0.96	1.25 ± 0.86	5.88	0.003
Sleep duration	0.31 ± 0.62	0.29 ± 0.68	0.38 ± 0.64	0.27 ± 0.58	0.83	0.436
Habitual sleep efficiency	0.15 ± 0.43	0.05 ± 0.29	0.26 ± 0.50	0.14 ± 0.43	3.93	0.021
Sleep disturbances	1.28 ± 0.54	1.09 ± 0.48	1.25 ± 0.49	1.39 ± 0.58	5.96	0.003
Use of sleeping medication	0.11 ± 0.53	0.09 ± 0.48	0.09 ± 0.41	0.14 ± 0.61	0.18	0.832
Daytime dysfunction	0.80 ± 0.81	0.78 ± 0.73	0.83 ± 0.59	0.80 ± 0.82	0.06	0.939

Table 4. Association of psychological disturbances and sleep quality

No.	Variables	Subcategories	Sleep Quality		X^2 value	p-value
			Good sleep quality n (%)	Poor sleep quality n (%)		
1.	Depression	No any depression status	135 (63.4)	78 (36.6)	4.499	0.034

		Possible depression of varying severity	14 (43.8)	18 (56.3)		
2.	Anxiety	No anxiety	136 (64.2)	76 (35.8)	10.799	0.013
		Mild level anxiety	11 (50.0)	11 (50.0)		
		Moderate level anxiety	2 (22.2)	7 (77.8)		
		Severe level anxiety	0 (0)	2 (100)		

Discussion

Quality sleeping is a substantial requirement during the pregnancy to maintain physical, psychological well-being of a mother and well-being of the fetus. Therefore present hospital based, cross-sectional study was conducted to determine the prevalence of poor sleep quality, compare difference of sleep quality in each trimester, psychological disturbances and other factors affecting for sleep quality among antenatal mothers.

In the current study, the mean age of years was comparable with several other studies carried out in developing [10] and developed countries. The monthly family income of majority of mother was lower (<LKR 50,000.00) than the nominal Sri Lankan mean house hold income of LKR 62237.00 [11] which may be attributed to the fact that most women in the study were unemployed.

Sleep quality was assessed by Pittsburgh Sleeping Quality Index, which is the validated tool for Sri Lanka and it helps to assess sleep quality by using seven components of sleep process over a one month period. In present study, two-third of study subjects had good sleep quality during first trimester. Findings of this study are in agreement with Ethiopian study which was conducted among 415 pregnant mothers. Ethiopian study also has used similar study tool, Pittsburgh sleep quality index as present study. Another study in Finland which was carried out with 78 pregnant mothers has got almost similar results even though they used Basic Nordic Sleep Questionnaire to collect data [12]. The plausible explanation may be that the

participants in these studies were in similar age category, economical studies and BMI level as important parameters.

Sleep quality can be affected by several factors. In a more recent study in China has reported that increased maternal age and gestational age are important determinants of sleep quality [5]. In present study, above factors were also identified as determinants. However, another, Ethiopian study has found multiparity as another determinant addition to the above factors [8]. Cultural differences of two countries may be the reason for this difference. Cultural differences of two countries may be the reason for this difference.

Quality of sleeping also varying within three trimesters of pregnancy due to fluctuation of hormonal level throughout the body. Mainly, Melatonin hormone helps for improve sleep level by regulating the circadian rhythms of several biological functions, including the sleep-wake cycle. According to the present study, women in third trimester experienced higher score of global PSQI, subjective sleep quality, sleep latency, habitual sleep efficiency, sleep disturbances and significantly longer sleep latency than women in other two trimesters. Further, good sleep quality had gradually decreased from first trimester to the third trimester. The findings are comparable with Taiwan study which was conducted with 400 antenatal mothers found that sleep latency, subjective sleep quality, and habitual sleep efficiency were significantly different by trimester and women in their third trimester had significantly longer sleep latency than women in their first trimester. Hence, women in the third trimester also had significantly lower habitual sleep efficiency and subjective sleep quality than second trimester [13]. In present study, mean sleep latency score was 1.06 ± 0.89 respectively. Almost similar results found in Iran study among 605 pregnant women, that mean sleep latency score and mean score of sleep disturbances gradually increased with gestational age [14]. These findings may be attributed to the similar physiological changes during pregnancy. It is known that, physical and mechanical factors such as frequent urination at night, very active baby, heart burn sensation and increased level of estrogen and oxytocin might be reason for increased sleep disturbance during pregnancy.

Anxiety and depression are common psychological disturbances during pregnancy and postpartum due to the fluctuation of maternal hormones [5]. Generalized anxiety disorders – 7 questionnaire is validated tool for Sri Lanka which can be used to assess the anxiety of general population. Edinburgh Postnatal Depression Scale is the questionnaire which is widely validated tool was used to assess the level of depression among mothers. Results of

this present study are in line with several studies [15] around the world. Poor sleep quality is experienced by more than a half of pregnant women who have possible depression of varying severity [10]. These finding may be attributed to the hidden fear of the impending birth of the child in women who experience pregnancy for the first time. This might be enhance with cultural background in our country, some pregnancy related myths such as whole baby care process belongs to the mother, knowledge and experience level of senior members of a family about pregnancy.

From this study, advanced maternal age, gestational age, prenatal depression and anxiety are detected to be determinants of sleep quality during pregnancy period. This finding was confirmed by a China study has found that the significant contributors of sleep quality for pregnant women were prenatal depression, age and gestational age [5]. Also similar results has found in Ethiopian study mentioned that advanced age, increased gestational age and multiparty are found to be determinants of poor sleep quality in pregnant women [8]. It is clear that our results are more similar to data published by other authors worldwide and may be an important voice in worldwide analysis of sleep quality during pregnancy.

The strength of our study is provide more detailed information to explain sleep quality with efficiently using limited amount of cost. Maintain anonymity and self-administered questionnaire might have promoted the honesty of the answers. To our knowledge, there is no other published research about this study area in Sri Lanka. However, limitations of the study should have been acknowledged. Though, the analyzed data are derived from a self-administered questionnaire, some questions had to be explained to some mothers. It could be the cause of recall bias. Though, validated questionnaires are limited in Sinhala language, study group always use the pretested translated questionnaire to minimize the effect of this limitation. Further, in this study it was not assess how disease conditions affect to the sleep quality during pregnancy period due to limited time and practical constrain.

Conclusions

This study showed, the prevalence of poor sleep quality among pregnant women was 39.2%. Advanced maternal age, gestational age, prenatal depression and anxiety were predominant determinants of sleep quality during pregnancy period. Good sleep quality had gradually decreased from first trimester to the third trimester. Women in third trimester

experienced higher habitual sleep efficiency, sleep disturbances and significantly longer sleep latency than women in other two trimesters. Among major components of quality sleeping according to the PSQI, subjective sleep quality, sleep latency, habitual sleep efficiency and sleep disturbances showed that significant difference around three trimesters. It is expected that findings of this research would be helpful for health and social care policy makers when formulating guidelines and interventions regard to improving quality of sleep among pregnant women in Sri Lanka. It is recommended to plan that planning awareness programs and assess sleep quality throughout the pregnancy with the aim of enhancing the quality of sleep of pregnant women. Further, research team would like to recommend to develop a cross cultural validated tool to assess sleep quality among pregnant women in Sri Lanka. Since Sri Lanka has unique cultural believes.

Acknowledgements

The authors would like to acknowledge the mothers for their participation.

References

1. Astuti Y. The Factors Influencing Sleep Quality of Pregnant Women in Yogyakarta , Indonesia. 2019;15(IcoSIHSN):203–6.
2. National Sleep Foundation 2020.
3. Silvestri, R., & Aricò, I. (2019). Sleep disorders in pregnancy. *Sleep science (Sao Paulo, Brazil)*, *12*(3), 232–239. https://doi.org/10.5935/1984-0063.20190098.
4. Du, M., Liu, J., Han, N., Zhao, Z., Yang, J., Xu, X., Luo, S., & Wang, H. (2021). Maternal sleep quality during early pregnancy, risk factors and its impact on pregnancy outcomes: a prospective cohort study. *Sleep medicine*, *79*, 11–18. https://doi.org/10.1016/j.sleep.2020.12.040.
5. Yang, Y., Mao, J., Ye, Z., Zeng, X., Zhao, H., Liu, Y., & Li, J. (2018). Determinants of sleep quality among pregnant women in China: a cross-sectional survey. *The journal of maternal-fetal & neonatal medicine : the official journal of the European Association of Perinatal Medicine, the Federation of Asia and Oceania Perinatal Societies, the International Society of Perinatal Obstetricians*, *31*(22), 2980–2985.

https://doi.org/10.1080/14767058.2017.1359831.

6. Reichner C. A. (2015). Insomnia and sleep deficiency in pregnancy. *Obstetric medicine*, *8*(4), 168–171. https://doi.org/10.1177/1753495X15600572.
7. Mirghaforvand M, Mohammad-alizadeh-charandabi S, Zarei S. Women on the Other Side of Depression War and Poverty : Its Effect The Relationship Between and Sleep Quality in on the Health of Reproduction Iranian Pregnant Women. Aras Part Med Int Press. 2017;5(2):147–152.
8. Jemere T, Getahun B, Tadele F, Kefale B, Walle G (2021) Poor sleep quality and its associated factors among pregnant women in Northern Ethiopia, 2020: A cross sectional study. PLOS ONE 16(5): e0250985. https://doi.org/10.1371/journal.pone.0250985
9. Silva-Perez, L. J., Gonzalez-Cardenas, N., Surani, S., Etindele Sosso, F. A., & Surani, S. R. (2019). Socioeconomic Status in Pregnant Women and Sleep Quality During Pregnancy. *Cureus*, *11*(11), e6183. https://doi.org/10.7759/cureus.6183.
10. Smyka, M., Kosińska-Kaczyńska, K., Sochacki-Wójcicka, N., Zgliczyńska, M., & Wielgoś, M. (2020). Sleep Problems in Pregnancy-A Cross-Sectional Study in over 7000 Pregnant Women in Poland. *International journal of environmental research and public health*, *17*(15), 5306. https://doi.org/10.3390/ijerph17155306.
11. Sri Lanka Household Income and Expenditure Survey 2016.
12. Polo-Kantola, P., Aukia, L., Karlsson, H., Karlsson, L., & Paavonen, E. J. (2017). Sleep quality during pregnancy: associations with depressive and anxiety symptoms. *Acta obstetricia et gynecologica Scandinavica*, *96*(2), 198–206. https://doi.org/10.1111/aogs.13056.
13. Hung H, Tsai P, Ko S, Chen C. in Taiwanese Pregnant Women. 2013;(April):95–101.
14. Effati-daryani F, Mohammad-alizadeh-charandabi S. Evaluation of Sleep Quality and Its Socio-demographic Predictors in Three Trimesters of Pregnancy among Women Referring to Health Centers in Tabriz , Iran : A Cross- sectional Study. 2019;(098 51).
15. Yucel SC, Yucel U, Gulhan I, Ozeren M. Sleep quality and related factors in pregnant women. 2015;(March).
