# Supplementary material for: Determinants of sleep quality among pregnant women in a selected institution in the Southern province, Sri Lanka
Source: PLoS One. 2024 Jul 18;19(7):e0305388. doi: 10.1371/journal.pone.0305388 (PMC11257308; doi:10.1371/journal.pone.0305388)
Supplement: Attachment — Submitted filename: response to reviewers4.26.docx [file pone.0305388.s007.docx]

**PONE-D-24-00504**

**Determinants of sleep quality among pregnant women in teaching hospital mahamodara, Galle, Sri Lanka**

**Response to the reviewers’ comments**

| **Comment** | **Response to reviewers’ comments** | **Line and page numbers** |
| --- | --- | --- |
| ***Editor*** | | |
| **Abstract Formatting:**  Ensure that your abstract strictly adheres to our Instructions for Authors' formatting guidelines. | ***We would like to thank the editor and reviewers for their valuable comments to robust the quality of the manuscript.***  ***We ensured that the abstract strictly adhered to the instructions for authors’ formatting guidelines.*** | Please refer to the revised introduction  Page 2-3  Lines 57 - 110 |
| **Supplementary File:**  Kindly include the supplementary (like questioner / consent/assent file as requested). | ***Supplementary files have been attached as requested.***  ***Supplementary file 1-6*** | 503 - 509 |
| **Introduction**:  Provide a clearer explanation of the research gap and emphasize the significance of the study in the introduction. | When following the recent literature, it was found that poor sleep quality enhances the 20% chances of undergoing lower segment caesarian sections and prolonged labor. Further, it is a potential risk factor for mental disorders during perinatal and postnatal period especially depression and it could lead to postpartum psychosis.  The nature of the sleep during pregnancy can be varied. These changes may be unique to each trimester since during these three trimesters, specific physiological and psychological changes occur relevant to each trimester.  Although poor sleep quality has a high prevalence and related complications, still it is a neglected and hidden factor among healthcare providers and health policymakers, especially in middle-income countries.  Several studies have been conducted in Western countries to assess sleep qualities and determinants during the pregnancy period, but it is a new field for Asian countries as well as for low and middle-income countries including Sri Lanka.  Therefore, we have included the below paragraphs to the introduction  ***‘Poor sleep quality enhances the 20% chances of undergoing lower segment cesarian sections and prolonged labor. Further, it is a potential risk factor for mental disorders during perinatal and postnatal periods especially depression and it could lead to postpartum psychosis.’***  ***‘The nature of the sleep during pregnancy can be varied. These changes may be unique to each trimester since during these three trimesters, specific physiological and psychological changes occur relevant to each trimester.***’  The last paragraph was modified as below.  ‘***Poor sleep quality has a high prevalence and related complications throughout the pregnancy, but it is a neglected and hidden factor among healthcare providers and health policymakers, especially in middle-income countries.***  ***Although several studies have been conducted in Western countries to assess sleep qualities and determinants during the pregnancy period, still it is a new field for Asian countries as well as for low and middle-income countries including Sri Lanka. Further, none of the reported studies in the South Asian region had compared the differences in sleep qualities and similar results among three trimesters and neither of them had identified associated factors for the poor quality of sleep during each trimester.’*** | 57 - 110 |
| **Source of Tool/Questionnaire:**  Specify the source of the tool/questionnaire used in your study. | Data collection and management (Materials and Methods section) | 145 - 174 |
| **Reliability and Validity:**  Outline the steps taken to assure the reliability and validity of your data. | To enhance the reliability and validity of the study,  several standard steps were taken throughout the study.  The appropriate sample was taken to the study with considering the 10% non-respondent rate to enhance the validity of the results.  The self-administered questionnaire which consisted of four parts was used to collect data.  Part A-Self developed part to assess socio-demographic data and pregnancy-related information.  Part B- Cross-cultural validated questionnaires were used to collect data for our study. Sinhala version of the validated Pittsburg Sleep Quality Index (PSQI) tool was used to assess the quality of sleep.  Part C-Sinhala version of the Edinburgh postnatal depression scale which was a widely validated tool to assess depression throughout the pregnancy and postpartum was used to identify the depression status.  Part D- Sinhala version of the General Anxiety Disorders 7 (GAD 7) scale is used to assess the anxiety status of the mothers. However, GAD 7 is not a cross-cultural validated tool hence content and face validity were assessed before applying it.  This questionnaire was a pretest with ten pregnant mothers in another setting in the Galle district to prevent participants' contamination.  To reduce the socially desirable bias and subjective bias, participants were asked to complete this questionnaire by themselves. However, trained investigators were there to give any assistance. | 145 - 174 |
| **Operational Definition and Measurement:**  Please provide clarification on how you measure the operational definitions. Include specific details such as the number and type of questions used to assess factors like sleep quality and anxiety." | Sleep quality, depression, and anxiety were identified as operational definitions of this study.  All the details of measuring operational definitions were included in the data collection and management section as below.  ‘*Sleep quality- Sleep quality was assessed by the Pittsburg Sleep Quality Index (PSQI). It was a cross-cultural validated tool for Sri Lankans with good internal consistency (Cronbach’s alpha=0.85). It consisted of 19 self-evaluated questions under seven components i.e. subjective sleep quality, sleep latency, sleep duration, habitual sleep efficiency, sleep disturbances, use of sleep medications, and daytime functions. These questions are scored to get a total mark range of 0-21 and a higher scale shows severe difficulty in all areas.*  *Depression status Depression status were assessed by the Edinburgh postnatal depression scale (EPDS). It is also a validated questionnaire for the Sri Lankan antenatal mothers (Cronbach’s alpha=0.95). It consists of 10 questions. Each answer is given a score from 0 to 3. The maximum score is 30 and higher indicates severe depressive status.*  *Anxiety level- Anxiety level was screened by General Anxiety Disorder 7 scale (GAD-7) .It consists of 7 items and each item was rated by 4 point Likert scale. Though it is a widely validated tool in several countries, still it is not a validated tool in Sri Lanka. Hence content and face validity of this questionnaire were assessed before applying it.* | 176 – 184  145 - 174 |
| **Categorization of Continuous Variables:**  Ensure that all continuous variables in the results section are categorized in a standard way or adapted from similar previous literature. For instance, the response to the question ‘How many hours of actual sleep did you get at night?’ was counted twice in the second and third columns. Please revisit this issue. | We regret the mistake that happened here. Previously, we categorized our continuous variables in the results sections in a standard way. But after referring to similar previous literature (Smyka et al., 2021) categorical variables have been slightly modified to enhance the clarity of the results.  ***(Smyka, M., Kosińska-Kaczyńska, K., Sochacki-Wójcicka, N., Zgliczyńska, M. and Wielgoś, M., 2021. Sleep quality according to the Pittsburgh Sleep Quality Index in over 7000 pregnant women in Poland. Sleep and biological rhythms, 19(4), pp.353-360.)*** | 252 – 253  Table 4 |
| **Limitations and Strengths:**  Include a section on limitations and strengths to provide a comprehensive study overview. | More limitations and strengths of this study were highlighted in the last paragraph of the discussion below  There are several limitations that we have to highlight in our study. This study was conducted in a single setting in the Galle district in Sri Lanka with one ethnic group, hence some generalizability issues could be found.  However, the respondent rate of this study is 100% of this study and all parts of the questionnaire were validated or pretested. Therefore, the validity and reliability of the study were at an optimal level which could be considered as one of the strengths of our study. Further, participants filled out the questionnaire by themselves. Hence, Subjective bias and socially desirability may probably be minimized.  This study may probably be the first published study not only in Sri Lanka but also in South Asia which leads future guides for healthcare workers to take actions to enhance the quality of the pregnant period and to carry out future studies with larger scales. | 347 - 370 |
| **Tables and Figures:**  Adhere to the guidelines regarding tables and figures. | All tables have been reformatted and labeled after considering journal guidelines carefully. |  |
| **Reviewer 1** |  |  |
| Data collection and management: tools validity etc are missed | Data collection and management subtitle was added as suggested by the reviewer. All the details of tool validations are explained in this section below.  ‘Sleep quality- Sleep quality was assessed by the Pittsburg Sleep Quality Index (PSQI). It was a cross-cultural validated tool for Sri Lankans with good internal consistency (Cronbach’s alpha=0.85). It consisted of 19 self-evaluated questions under seven components i.e. subjective sleep quality, sleep latency, sleep duration, habitual sleep efficiency, sleep disturbances, use of sleep medications, and daytime functions. These questions are scored to get a total mark range of 0-21 and a higher scale shows severe difficulty in all areas.  Depression status - Depression status were assessed by the Edinburgh Postnatal Depression Scale (EPDS). It is also a validated questionnaire for the Sri Lankans antenatal mothers (Cronbach’s alpha=0.95). It consists of 10 questions. Each answer is given a score from 0 to 3. The maximum score is 30 and higher indicates severe depressive status.  Anxiety level- Anxiety level was screened by General Anxiety Disorder 7 scale (GAD-7) .It consists of 7 items and each item was rated by 4 point Likert scale. Though it is a widely validated tool in several countries, still it is not a validated tool in Sri Lanka. Hence content and face validity of this questionnaire were assessed before applying it. | 145 - 174 |
| Line 130: short form used with? meaning | O/L refers to “Ordinary Level” examination | 221 |
| There are just 15 references, seems lack of sufficient evidences to support the research | Agreeing with the reviewer's comment, we elaborate on the overall article by using more literature to enhance the quality of the article. Newly added references were appropriately added to the reference list. | 405 - 499 |
| Line 209-210: sentence repeated, cultural difference should be further elaborated | Apologize for the mistake. Removed the repeated sentence and the statement of cultural difference. To elaborate on the clarity of the paragraph and the cultural differences, fifth paragraph of the discussion was revised as below.  Sleep quality can be affected by several sociodemographic and other pregnancy-related factors. In the present study, a statistically significant association was observed between gestational age and poor sleep quality among pregnant mothers, showing that sleep quality decreases as pregnancy continues. This is in parallel with the findings of studies conducted in China [4], and Ethiopia [17]. This may be attributed to hormonal changes, increased maternal anxiety, and fetal movement in the final trimester of the pregnancy. | 288 - 345 |
| Line 238-241, lack of reference | Lacking references were added. | 332 |
| Result of similar studies in Asia and Africa are used, studies in Europe and America are also suggested to be used and compare | Agreeing with the reviewer's comments, European and American similar studies also referred to and added literature in the suitable areas. | 322 – 325  337 - 340 |
| Line 272-274, please further elaborate the suggestion with example and reference | Suggestions were elaborated with examples as below.  It would be better to use a screening tool to assess the sleep quality of mothers in clinical practices to take necessary measurements to enhance sleep quality of pregnant mothers as suggested by a recent study (17) to robust the quality of life during pregnancy.  As suggested by Smyka et al., we also suggested to discuss sleep hygiene, risk factors for poor sleep, and the importance of sleep during pregnancy with mothers and family members.  *(Smyka, M., Kosińska-Kaczyńska, K., Sochacki-Wójcicka, N., Zgliczyńska, M. and Wielgoś, M., 2021. Sleep quality according to the Pittsburgh Sleep Quality Index in over 7000 pregnant women in Poland. Sleep and biological rhythms, 19(4), pp.353-360*.)  A large-scale study could be suggested to conduct with different ethnic groups and cultures to enhance the validity of the study.  Further, as suggested by the reviewer 3, following suggestions were included.  ‘It is suggested to tailor these findings to relevant government agencies and public health policies to plan awareness programs to enhance maternal health at the primary care level and to enhance the sleep quality throughout the pregnancy.’ | 387 - 394 |
| **Reviewer 2** |  |  |
| The study investigated determinants of sleep quality among pregnant women in Sri Lanka, highlighting the scarcity of research in this area, particularly within the Sri Lankan context. The manuscript is well-organized, highlighting the study's significance to Sri Lanka and the maternal health context. Study objectives are well outlined. Although some study limitations are recognized, further improvements and clarifications are necessary to enhance the manuscript's overall contribution to public health literature. |  |  |
| **Introduction**: Authors to consider expanding on the introduction to include more recent studies from around the world to provide a comprehensive overview of evidence on sleep quality among pregnant women and its impact on pregnancy outcomes. | After considering the editor and all reviewers’ comments, the introduction was modified as below.  “***‘Poor sleep quality enhances the 20% chances of undergoing lower segment cesarean sections and prolonged labor. Further, it is a potential risk factor for mental disorders during perinatal and postnatal periods especially depression and it could lead to postpartum psychosis.’***  ***‘The nature of the sleep during pregnancy can be varied. These changes may be unique to each trimester since during these three trimesters, specific physiological and psychological changes occur relevant to each trimester.***’  The last paragraph was modified as below.  ‘***Poor sleep quality has a high prevalence and related complications throughout the pregnancy, but it is a neglected and hidden factor among healthcare providers and health policymakers, especially in middle-income countries.***  ***Although several studies have been conducted in Western countries to assess sleep qualities and determinants during the pregnancy period, still it is a new field for Asian countries as well as for low and middle-income countries including Sri Lanka. Further, none of the reported studies in the South Asian region had compared the differences in sleep qualities and similar results among three trimesters and neither of them had identified associated factors for the poor quality of sleep during each trimester.’*** | 57 - 110 |
| The methodology appears robust, employing a descriptive cross-sectional design with a reasonable sample size calculated based on acceptable methodology.  However, clarification on data collected using PSQI, EPDS, GAD-7 would be helpful to the reader. For example, it is not clear if social factors influencing sleep quality such as family support, and partner relationship characteristics, and drug and alcohol use were collected /assessed. | All the information related to the questionnaires was explained under the “ data collection and management tool” below  “Sleep quality- Sleep quality was assessed by the Pittsburg Sleep Quality Index (PSQI). It was a cross-cultural validated tool for Sri Lankans with good internal consistency (Cronbach’s alpha=0.85). It consisted of 19 self-evaluated questions under seven components i.e. subjective sleep quality, sleep latency, sleep duration, habitual sleep efficiency, sleep disturbances, use of sleep medications, and daytime functions. These questions are scored to get a total mark range of 0-21 and a higher scale shows sever difficulty in all areas.  Depression status- Depression status were assessed by the Edinburgh postnatal depression scale (EPDS). It is also a validated questionnaire for the Sri Lankans antenatal mothers (Cronbach’s alpha=0.95). It consists of 10 questions. Each answer is given a score from 0 to 3. The maximum score is 30 and higher indicates severe depressive status.  Anxiety level- Anxiety level was screened by General Anxiety Disorder 7 scale (GAD-7) .It consists of 7 items and each item was rated by 4 point Likert scale. Though it is a widely validated tool in several countries, still it is not a validated tool in Sri Lanka. Hence content and face validity of this questionnaire were assessed before applying it.  Social factors, family support, relationship characteristics and drug use were assessed by using PSQI questionnaire. | 145 - 174 |
| **Questionnaire** – a local language and English was used, yet most respondents only had O/L or below, can the authors clarify why trained data collectors were not considered to administer questionnaires? | Questionnaires were prepared in both languages but they were allowed to select the most convenient language to fill the questionnaire. Further, all the questionnaires were prepared in simple language without medical jargon. All were pretested with similar subjects in a different setting and all the ambiguous statements and interpretation errors were corrected. Though most participants had been educated up to the ordinary level or below, Sri Lankan literacy level is 92.25% [15]. Hence, we assumed that they could understand these simple statements. However, trained investigators were there to give any assistance.  To enhance the validity and to reduce the socially desirable bias and subjective bias participants were asked to complete this questionnaire by themselves. |  |
| Moreover, how depression and anxiety were assessed needs clarification. | The section on data collection and management tools is explained as below.  ‘Depression status- Depression status were assessed by the Edinburgh postnatal depression scale (EPDS). It is also a validated questionnaire for the Sri Lankan antenatal mothers.  Anxiety level- Anxiety level was screened by the General Anxiety Disorder 7 scale (GAD-7). It consists of 7 items and each item was rated on 4 point Likert scale.’ | 186 - 194 |
| Although study limitations have been acknowledged, clarifying potential bias/limitation in the sampling process would enhance study credibility. For example, the manuscript did not outline if the influence of existing conditions likely to influence sleep quality was considered, or bias of any sort. | Limitations have been mentioned in the manuscript and highlighted in the last paragraph of the discussion below  There are several limitations that we have to highlight in our study. This study was conducted in a single setting in a Galle district in Sri Lanka with one ethnic group, hence some generalizability issues are there.  All the measurements were taken to reduce the potential biases such as social desirable bias, subjective bias etc.  We considered the below mentioned existing conditions that were likely to influence sleep quality. Their influences have been tabled in table 2  Maternal age  Level of Education  Professional engagement  Family income  Environmental factors  Maternal anxiety and depression | 347-370 |
| Authors to clarify study response rate, and further outline how incomplete questionnaires were dealt with. | All the information related to the response rate and incomplete questionnaire was included in the data collection and management section below  ‘All the respondents participated in the study voluntarily. Hence, the response rate of the study was 100%. The incomplete questionnaires were removed from the study and a new response was collected until the sample size achieved.’ | 172 - 174 |
| Provide background information on study the setting, such as population characteristics etc., how many pregnant women are treated per year. | Background information on study the setting was provided under the sub-title of study setting and study population below  ‘Teaching Hospital Mahamodara, Sri Lanka is the only maternity teaching hospital in Southern Province, Sri Lanka. It consisted of three antenatal wards and three separate antenatal clinics attached to these wards. Approximately 3000 antenatal mothers registered to the clinic for a month and around 600 antenatal mothers were admitted to the antenatal wards. | 114 - 119 |
| Did the authors target patients enrolled at the center during a certain period, considering data was collected in a single month, please clarify how the list was drawn up. | There was a separate clinic register for all antenatal mothers. The clinic days of the week were divided into three antenatal wards and there was a separate register to admit the antenatal mothers to the wards. Based on these registers we recruited mothers by a systematic random sampling technique.;individuals who belonged to the even number. To ensure the diversity of the sample, mothers were selected from both antenatal clinics and wards. Fifteen to twenty-five mothers per day were selected and within 20 days sample size was achieved. | 136 - 143 |
| The results section mentions monthly income but population background regarding social factors such as living conditions, average household income, and employment trends in the area are not provided. Difficult to understand how the results would be interpreted in the absence of the aforementioned. | Thank you very much for the comment. We didn’t assess the living conditions of these mothers since living conditions can vary with sleeping problems.  Further, we had to use separate tools to assess the living conditions but it was practically difficult to spare more time since we had a very limited period. However, we identified it as a limitation and we mentioned it in the strength and limitation section  Further, when considering female labor force participation rate in Sri Lanka is 30 – 35% ([https://www.ilo.org/wcmsp5/groups/public/---asia/---ro-bangkok/---ilo-colombo/documents/publication/wcms_551675.pdf), during](https://www.ilo.org/wcmsp5/groups/public/---asia/---ro-bangkok/---ilo-colombo/documents/publication/wcms_551675.pdf),during) pregnancy it reduces further. The results of this study also showed that 73.1% of mothers were unemployed. The husband is the only breadwinner in the majority of families. Hence, the income reflects the average household income of the families. This information was added as monthly income in Table 1 and women's employment participation across Sri Lanka is low since they are unable or unwilling to engage in paid works. (<https://asiapacific.unwomen.org/en/digital-library/publications/2022/03/the-demand-for-women-workers-in-sri-lanka#:~:text=Most%20Sri%20Lankan%20women%20are,as%20un>). Hence, these factors were not considered.” | 347 – 370 |
| Authors to consider defining key concepts/terms such as A/L and O/L throughout the manuscript to improve clarity and other undefined terms. Majority of participants appear to have an education level of up to O/L but it is not clear what this means, and could authors consider merging categories with low participants, for example, categories up to A/L can be merged | All the key concepts/terms like O/L, A/L were defined under the ‘Result” section below  O/L refers to ‘Ordinary Level’ examination  A/L refers to ‘Advanced Level’ examination  Rephrase the sentence to enhance the clarity as below  ***“Majority of subjects were married (98.8%), unemployed (73.1%) and education up to Ordinary Level - O/L (43.3%) with monthly income between 30,000 – 40,000 rupees.”***  Merging some categories with low participants to enhance the validity of the results. | 232 – 233  220 - 224 |
| The results indicated that 39.2% of participants experienced poor sleep quality, while 60.8% enjoyed good sleep quality, with a mean global PSQI score of 4.8531 (SD = 2.3053). Most participants reported good sleep quality during the last month of their pregnancy, according to the PSQI results. Authors to consider referencing the specific tables where these data are drawn from for clarity (Lines 140 to 141). | Specific tables were appropriately referred to as the reviewer suggested below. | 239 – 240  Table 03  263 - 264 |
| To improve clarity and demonstrate a consideration of scientific rigor. When claiming a significant difference, the Authors explicitly outline the P-value (example Line 174), In addition, the Authors outline % with several respondents where reference Is made to the majority or frequency of key factors. | To improve the clarity of the results, P-value was added to all the relevant places.  Percentages were presented in all the needy places in the results. | 48 - 49  220 - 286 |
| The presentation of results is consistent, and the statistical analysis performed is appropriate. However, the study could be enhanced by additional analyses. Authors to consider stratifying analysis by trimester or gestational age and conduct logistic regression to adjust for confounders, reporting odds ratios (OR) and confidence intervals (CI), which will give scientific rigor to the paper. | As suggested by reviewers multinomial logistic regression was conducted | 284 - 285 |
| Given that gestational age showed significant variance, stratifying the analysis by gestational age could offer a deeper understanding of its impact on sleep quality. | After considering all the related factors multinomial logistic regression was conducted and results were presented in table 7 | 284 - 285 |
| Furthermore, the significance of maternal age (p-value = 0.001) suggests the need for logistic regression analysis to determine the odds ratio for poor sleep quality across different ages and trimesters. Highlighting these odds ratios would provide clearer insights into the risk factors associated with poor sleep quality during pregnancy. | After identifying the linear relationships with sleep quality, gestational age, age categories, depressive status and anxiety, Multinomial Logistic Regression was conducted. The results showed that good sleep is 3.156 higher in the first trimester than the other two semesters. Didn’t show any impact from other variables. The results included in table 6 | 284 - 285 |
| Additionally, exploring how education and social factors, including the number of children, affect sleep quality across trimesters could enrich the study's findings. | The suggested information was added to the table 7. However, we didn’t observe any association between sleep quality and these factors. | 284 - 285 |
| In the abstract, it is mentioned, "Gestational age and increasing maternal age were significantly associated with sleep quality" (Line 31). Also, see Lines 37-38. Indicating the p-value where significant differences are noted would be beneficial. | P-value added to the relevant points as suggested by the reviewer. | 48 - 49 |
| Line 30 states, "60.8 of participants had good sleep quality and did not experience depression and anxiety." Also, consider Lines 44-45. It is not clear what 60.8 refers to. | The sentence was rephrased to enhance the clarity as below  ‘The majority of participants (60.8%) had good sleep quality and they didn’t have either depressive symptoms (63.4%) or anxiety (64.2%). | 40 - 41 |
| Authors to consider tailoring recommendations to relevant government agencies, stakeholders, and public health policies based on the study findings for greater impact. | Highly appreciate the reviewer suggestion. We included this suggestion under the conclusion as below  “It is suggested to tailor these findings to relevant government agencies and public health policies to plan awareness Programme to enhance maternal health at the primary care level and to enhance the sleep quality throughout the pregnancy’. | 392 - 394 |
| Citation Consistency: It is noted in various sections that studies have been reported, but often only one or two studies are cited (Line 61). | Apologize for the mistake. All the incite references and the reference list has been updated with relevant references. | 405 - 499 |
| **Tables and Terminology**: Tables and their titles could be improved, and terms like F and P should be defined, particularly for Table 3. | Acknowledge the comments. Tables and their title were modified, F, P and all symbols in tables were defined as below  F- F-test  P-P value | 263 - 264 |
| **Ethics Discussion Repetition:** Ethics-related issues are mentioned in more than one section of the manuscript, specifically on Lines 102-103. | Details related to the ethical clearance was added to the “Ethical approval & consent to participate” section as below. All the repetitions were removed.  “Ethical clearance was obtained from the Ethical Review Committee, Faculty of Allied Health Sciences, University of Ruhuna (No: 59.11.2021). Informed written consent was taken from all participants after providing detailed information of purpose of the research study, advantages, results, confidentiality, and methods of the research.  “ | 213 - 218 |
| **Definition of Severe Psychiatric Illnesses**: The authors should clarify how severe psychiatric illnesses were defined, as mentioned in Lines 79-80. | This definition is provided under the “Eligibility criteria” as below  ‘who had severe psychological problems that are severely impaired on person’s ability to engage functional and occupational activities such as bipolar disorders, schizophrenia etc.” | 124 - 125 |
| **Recommendation**  This study has the potential to significantly contribute to maternal health and public health. However, to achieve this, major revisions are required. These should focus on expanding the methodology by clarifying details as outlined above, addressing study weaknesses and biases, enhancing the analysis, and broadening the background section to include pregnancy outcomes linked to sleep quality. Additionally, grammatical errors need correction. | Highly appreciate the reviewer's comments. The methodology section was modified according to the editor's and reviewers’ comments.  A separate paragraph was added to the background section to explain the link between pregnancy outcomes and sleep quality.  ‘***Poor sleep quality enhances the 20% chances of undergoing lower segment cesarian sections and prolonged labor. Further, it is a potential risk factor for mental disorders during perinatal and postnatal period especially depression and it could lead to postpartum psychosis.’***  ***‘The nature of the sleep during pregnancy can be varied. These changes may be unique to each trimester as during these three trimesters, specific physiological and psychological changes occur relevant to each trimester.***’  Grammatical errors were corrected by the professional English lecturer, Faculty of Allied Health Sciences, the University of Ruhuna. Mention these details in the acknowledgment section as below  “We would like to acknowledge Ms. L. H. W. Dilprabha, lecturer, Faculty of Allied Health Sciences, University of Ruhuna, Sri Lanka for correcting the language errors. | 53 - 82 |
| **Reviwer3** |  |  |
| The authors' contribution, did the first author only contribute by collecting data? Was the first author involved in writing the manuscript? | All the authors’ contributions were included according to the guidelines on the first page as mentioned below  ‘The author contributed to all of the following: conceptualization and design of the study, collecting data, formal analysis and interpretation of data, and writing the manuscript.’ | Title Page |
| On the abstract, the first sentence is the same as the one in a paper by Anbesaw found on: <https://bmcpsychiatry.biomedcentral.com/articles/10.1186/s12888-021-03483-w>. May you please rephrase it?  On the same abstract, line 30, you have a value of 60.8, please indicate the units.  And on data analysis, what variables were involved, and what type of data was collected. | Regret for the mistake and it was rephrased as below  Sleep is a vital requirement during pregnancy for the betterment of the fetus and the mother. Sleep quality can vary due to pregnancy-specific psychological and physiological changes. To introduce a tailored programme to enhance the sleep quality of mothers, it is paramount to assess the sleep quality and determinants of sleep. Therefore, this study aimed to assess the determinants of sleep quality among pregnant women in a selected institution in the Southern province of Sri Lanka.’  The line 30 was corrected as below  ‘The majority of participants (60.8%) had good sleep quality and they didn’t have either depressive symptoms (63.4%) or anxiety (64.2%).’  The following details were added to the data analysis.  Below variables were involved and both continuous and categorical data were collected as required.  ‘Maternal sleep quality, socio-demographic data and gestational age, maternal depression and anxiety.’ | 23 - 28  40 - 42 |
| On methods, was the study only descriptive or also analytical? On sample size, line 92, is it not fine to mention the name of clinic? What is a self-developed questionnaire? Line 103, on ethical consideration should be deleted because there is a full sub section on ethical considerations. It is indicated that simple random sampling was used but you also indicate that the even numbers were selected. This is not simple random sampling. Please indicate how variables were measured. Were there scales? What type of data were used? | Acknowledge the suggestion and the study design was modified as descriptive-analytical.  In Sri Lankan settings these clinics are commonly named as hospital antenatal clinics. Hence, we modified the sentence to include the word “hospital clinic” in a study setting  We regret the mistake. It should be corrected as ‘self-administered’ instead of ‘self-developed’  All the repetitions related to the ethical clearance were deleted and all information was included in ‘the Ethical Approval & consent to Participate’ section.  We regret the mistake. It should be corrected as “systematic random sampling”  The information related to the variable measurements and the scales was in data collection and management section as below  “  Sleep quality- Sleep quality was assessed by the Pittsburg Sleep Quality Index (PSQI). It was a cross-cultural validated tool for Sri Lankans with good internal consistency (Cronbach’s alpha=0.85). It consisted of 19 self-evaluated questions under seven components i.e. subjective sleep quality, sleep latency, sleep duration, habitual sleep efficiency, sleep disturbances, use of sleep medications, and daytime functions. These questions are scored to get a total mark range from 0-21 and a higher scale shows sever difficulty in all areas.  Depression status- Depression status were assessed by the Edinburgh postnatal depression scale (EPDS). It is also a validated questionnaire for the Sri Lankan antenatal mothers (Cronbach’s alpha=0.95). It consists of 10 questions. Each answer is given a score from 0 to 3. The maximum score is 30 and higher indicates severe depressive status.  Anxiety level- Anxiety level was screened by the General Anxiety Disorder 7 scale (GAD-7) .It consists of 7 items and each item was rated by 4 point Likert scale. Though it is a widely validated tool in several countries, not a validated tool in Sri Lanka. Hence content and face validity of this questionnaire were assessed before applying it. | 114  116 – 117  155 - 156  213 – 218  140  145 - 174 |
| Line 105, the operational definitions should be included in the introduction and not stand alone.  On data analysis, the SPSS should be IBM.  Did you test for normality to confirm if data was normally distributed? Was the data suitable for ANOVA? | Operational definitions were included in the introduction as well as in a separate section  SPSS corrected as “ IBM SPSS” on data analysis  The normality of the data was checked by using the Kolmogorov–Smirnov test. P value > 0.05 was considered as normally distributed. After checking all the assumptions for the ANOVA in addition to the normal distribution of the data set, this test was used | 176 - 194  197 |
| On results, the words majority, most etc are subjective. It’s better to start with demographics then prevalence of poor sleep quality etc.  Table 2 has variables which look like questions taken directly from the questionnaire. Please check. | We also agree with the reviewer's comment. The results section started with socio-demographic data, followed by prevalence of sleep quality.  Table 4 was rechecked and modified to enhance the clarity of the data presentation. | 252 - 253 |
| Results on age show that age was measured as categorical data so where are the means coming from? Similar with other categorical variables. Please explained how these were measured. | Regret the mistake. There isn’t a mean value of the age. That sentence was removed from the results section. The relationships between continuous variables and categorical variables were checked by using ANOVA and the independent sample T-test after checking the normality of the continuous variables. | 226 - 227 |
| You have made claims on presence of statistical significant relationship without evidence. There is a mix up on presentation of results, you present results for Table one under a different health i.e. under Table 2 or 3. Any explanation on the Global PSQI score? | The results section was rearranged and repetitions were removed  Explain for the Global PSQI was provided under “operational definitions” as below  ‘The Pittsburg Sleep Quality Index (PSQI). It was a cross-cultural validated tool for Sri Lankans with good internal consistency (Cronbach’s alpha=0.85). It consisted of 19 self-evaluated questions under seven components i.e. subjective sleep quality, sleep latency, sleep duration, habitual sleep efficiency, sleep disturbances, use of sleep medications, and daytime functions. These questions are scored to get a total mark range of 0-21 and a higher scale shows sever difficulty in all areas. | 219 - 286 |
| On discussion, the first paragraph need to be deleted. It is important to start by answering the main research question, explain the answer and then back it up with other literature. May you please learn from other well written papers on how to write a good discussion section? | Acknowledge the given suggestion. The discussion was modified after considering the editor's and other reviewers' comments. | 288 - 345 |
| Reviewer 4 |  |  |
| The paper presents a contribution to the body of knowledge especially in the field of maternal and child health especially promoting mental health and psychological support for pregnant women by ensuring quality of sleep during pregnancy.  The structure of the paper is coherent and flows logically from the introduction to the conclusion.  The research undertaken is contextualized clearly. The researcher has studied and used appropriate number of bibliography sources used and quoted in the paper.  It is the evidence of the deep theoretical knowledge and very good orientation in the problem discussed in the paper.  The word processing of the paper is adequate. The using of different fonts and structure of the text is proper and helps the reader to better orientation in the text.  The paper is in a form suitable to the discipline. The format and literary presentation of the paper are satisfactory. Writing of the document is of professional standard. | Acknowledge the comment was given. |  |
